# A risk-based preoperative evaluation method and management strategy for removing intrauterine contraceptive devices in postmenopausal women

**DOI:** 10.3389/frph.2025.1576265

**Published:** 2025-08-25

**Authors:** Zijun Li, Yaqin Zheng, Min Liu, Linlin Pan

**Affiliations:** ^1^ Department of Gynecology, Longquan City People’s Hospital Affiliated to Lishui University, Lishui, Zhejiang, China; ^2^ Clinical Laboratory Centre, Longquan City People’s Hospital Affiliated to Lishui University, Longquan, Zhejiang, China; ^3^ Department of Gynecology, Zhejiang Quhua Hospital Affilated to Hangzhou Medical College, Quzhou, Zhejiang, China; ^4^ Department of Gynecology, Longquan Traditional Chinese Medicine Hospital, Lishui, Zhejiang, China

**Keywords:** intrauterine contraceptive devices (IUCDs), postmenopausal women, removal, risk scoring system for removal of IUCDs (RSSR-IUCDs), strategy, management

## Abstract

**Background:**

A large population of women have intrauterine contractive devices (IUCDs) as a result of China’s national family planning policy; this has created a significant economic burden and raised technological challenges related to the safe removal of IUCDs in postmenopausal women. It is very important to develop a risk scoring system for the removal of IUCDs (RSSR-IUCDs) to evaluate the preoperative risk of removal and offer management strategies for postmenopausal women.

**Methods:**

A systematic case retrospective analysis was conducted on 320 enrolled women who underwent IUCD removal surgery. After screening, stratifying high-risk factors, and final multifactor Logistic Stepwise Regression Analysis, a model named RSSR-IUCDs was constructed. It was verified using the Hosmer-Lemeshow test and a Receiver Operating Characteristic (ROC) curve was plotted to further analyze the predictive accuracy of IUCD removal failure.

**Results:**

Seven high-risk factors were finally selected, namely duration of menopause, IUCD retention time, a history of uterine surgery, the shape of the IUCD, IUCD position, uterine size, and uterine position. The RSSR-IUCDs was developed and demonstrated goodness of fit (χ^2^ = 236.558, *P* = 0.000). The score range of RSSR-IUCDs (Minimum–Maximum) was 0–40. The ROC curve of RSSR-IUCDs demonstrated that the ideal cutoff value was 20 points and the sensitivity and specificity of an initial failure to remove an IUCD were 69.60% and 95.60%, respectively.

**Conclusions:**

The RSSR-IUCD is a scientific, reasonable, and feasible evaluation system which is expected to become a guiding scoring system in accordance with clinical practice for postmenopausal women before the removal of IUCDs.

## 1 Introduction

The intrauterine contraceptive device (IUCD) is a safe and effective method for delaying or spacing pregnancies and is available for free or at low cost through global public health systems (1). Around 14.3% of women globally use this contraceptive method, and a 41% use rate of women in China means China has the highest IUCD prevalence worldwide (2). Between 1982 and 1990, an average of approximately 10 million women nationwide had an IUCD placed each year because of China's one-child fertility policy (3). However, approximately one-fourth of women older than 45 years have not yet had their IUCDs removed, largely because they are unaware of when an IUCD should be removed (3, 4) and have insufficient health education to advise them on the on-time removal of IUCDs at menopause. For postmenopausal women, 16.77% (170/1,014) did not undergo surgery to remove their IUCD (4). Given China's family planning policy, the large population of women with IUCDs poses a significant economic burden and technological challenge for the safe removal of IUCDs in postmenopausal women. Ultrasound evaluation is crucial to determine the IUCD position and assess any complications (5). However, many types of IUCDs pose a challenge for ultrasound evaluation, especially for many Chinese women in county-level hospitals who are unaware of the type of IUCDs they had placed (4). Numerous studies (3, 4) and reports have shown that there are many cases of IUCD fracture (6), secondary displacement (7), and perforation (6, 8, 9) caused by blind attempts to remove IUCDs in gynecological clinics. Given the above, the removal of IUCDs in postmenopausal women is currently a necessary skill for gynecologists, and it is necessary to standardize preoperative risk assessment and shunt management. Therefore, it is necessary and urgent to construct a risk-based preoperative evaluation model and management strategy for removing IUCDs in postmenopausal women.

## 2 Materials and methods

### 2.1 Study population

This study retrospectively analyzed 480 postmenopausal women who underwent IUCD removal by gynecologists with different professional titles at two county-level hospitals in Longquan and one city-level hospital in Quzhou, Zhejiang Province, between January 2020 and January 2024. The definition of menopause is menstruation that has stopped for at least one year according to the standards of the International Menopausal Association. The exclusion criteria were as follows: incomplete case data information including missing data, pharmacotherapeutically induced menopause in women with breast cancer, and women with severe acute or chronic disorders (such as acute or chronic heart failure or sinus bradycardia) who cannot afford direct removal surgery. Ethics approval was obtained from the Institutional Review Board of Longquan People's Hospital Affiliated to Lishui University (IRB-LPHALU-20220115; June 15, 2022). The study was performed in accordance with the ethical standards laid down in the 1964 Declaration of Helsinki and its later amendments. The necessity of informed consent was waived.

### 2.2 Data collection

This study comprehensively collected information on six aspects of postmenopausal women before IUCD removal. First, inclusion of general information such as patient age, body mass index (BMI), duration of menopause, number of abortions, parity, and education level. Second, a history of uterine surgery, including cesarean section (CS), myomectomy, and cervical conization or loop electrosurgical excision procedure (LEEP) was collected. Third, preoperative IUCD-related information, including the timing of IUCD placement, preoperative patient's awareness of the type of IUCDs, IUCD placement time, and the presence or absence of tail fibers was collected. Fourth, the results of preoperative imaging evaluation of IUCDs, including the presence or absence of metal components, and the shape and position of IUCDs were collected. Imaging evaluation mainly includes routine abdominal ultrasound (TAS) or transvaginal ultrasound (TVS) and necessary pelvic x-ray examination, all of which are carried out by professional ultrasound physicians and radiologists. Fifth, the evaluation results based on gynecological examination, including uterine size, uterine position, cervical size, and condition of cervical canal opening were collected. Lastly, the success or failure outcome of removing IUCDs, including the outcome of removing an IUCD for the first time and the results of ultrasound-guided second IUCD retrieval, were collected. Some conceptual issues involved in this study are defined in a standardized manner based on the clinical practice of the Chinese Society of Family Planning (CSFP) and Chinese Medical Association (CMA) (10), including the normal position of IUCD, IUCD embedment, and rupture, except for some pretreatments of perioperative patients including the routine use of prostaglandin drugs to promote cervical maturation. The normal position of the IUCD is the center of uterine cavity between the uterine fundus and the internal opening of cervical anatomy. IUCD embedment is defined as the myometrial penetration of the IUCD without serosal extension. The definition of IUCD rupture is that the integrity of the IUCD does not exist or it is fragmented or divided into several parts. The definition of normal uterine size is as follows: the uterus can be palpated in gynecology, with a maximum diameter of approximately 5.0–7.0 cm. The definition of a severely atrophied uterus is as follows: a gynecological palpable uterus with significant atrophy and reduction, with a maximum diameter of approximately 3.0–4.0 cm and a walnut-like shape. Moderate atrophy is a size between the two. The definition of a normal cervical size is from 2.5 cm to 3.0 cm, mild cervical atrophy is a cervical size from 2.0 cm to 2.5 cm, severe cervical atrophy is a size less than 1.0 cm, and moderate atrophy is a size between mild and severe atrophy.

### 2.3 Statistical analysis

Statistical analysis was performed with SPSS software (version 19.0, Chicago, IL, USA). First, different statistical processing methods were used to screen out individual risk factors. Nonnormally distributed data were presented as the median (M) and interquartile range (Q) and intergroup comparisons were performed using the Mann–Whitney *U*-test or the Kolmogorov–Smirnov test. The mean ± standard deviation was used for continuous quantitative data, and an independent sample t-test was used for intergroup differences. The rates of categorical data were compared using the Chi square (χ^2^) test or Fisher's exact test. Differences were statistically significant with *P* < 0.05. Spearman correlation analysis was used to examine the correlations of various factors: when the correlation coefficient between two variables was greater than 0.5 (*r* > 0.5), the excluded variables were carefully selected based on clinical practice and statistical principles. multivariable logistic regression analysis was performed on the risk factors included in the initial screening (inclusion condition: *P* < 0.05). After stratifying the high-risk factors (low risk, medium risk, high risk) based on initial screening risk factors, further multivariable Binary Logistic Stepwise Regression Analysis (Forward LR) was conducted and a model named RSSR-IUCDs was developed according to the minimum regression coefficient ratio method (rounded to the nearest integer). Finally, the Hosmer-Lemeshow Fit test was used to evaluate the fitness of the model, and its ROC (Receiver Operating Characteristic) curve was plotted to further analyze its clinical predictive accuracy of the first IUCD removal failure.

## 3 Results

### 3.1 The grouping and outcomes of 320 patients who underwent IUCD removal

**Figure 1:**
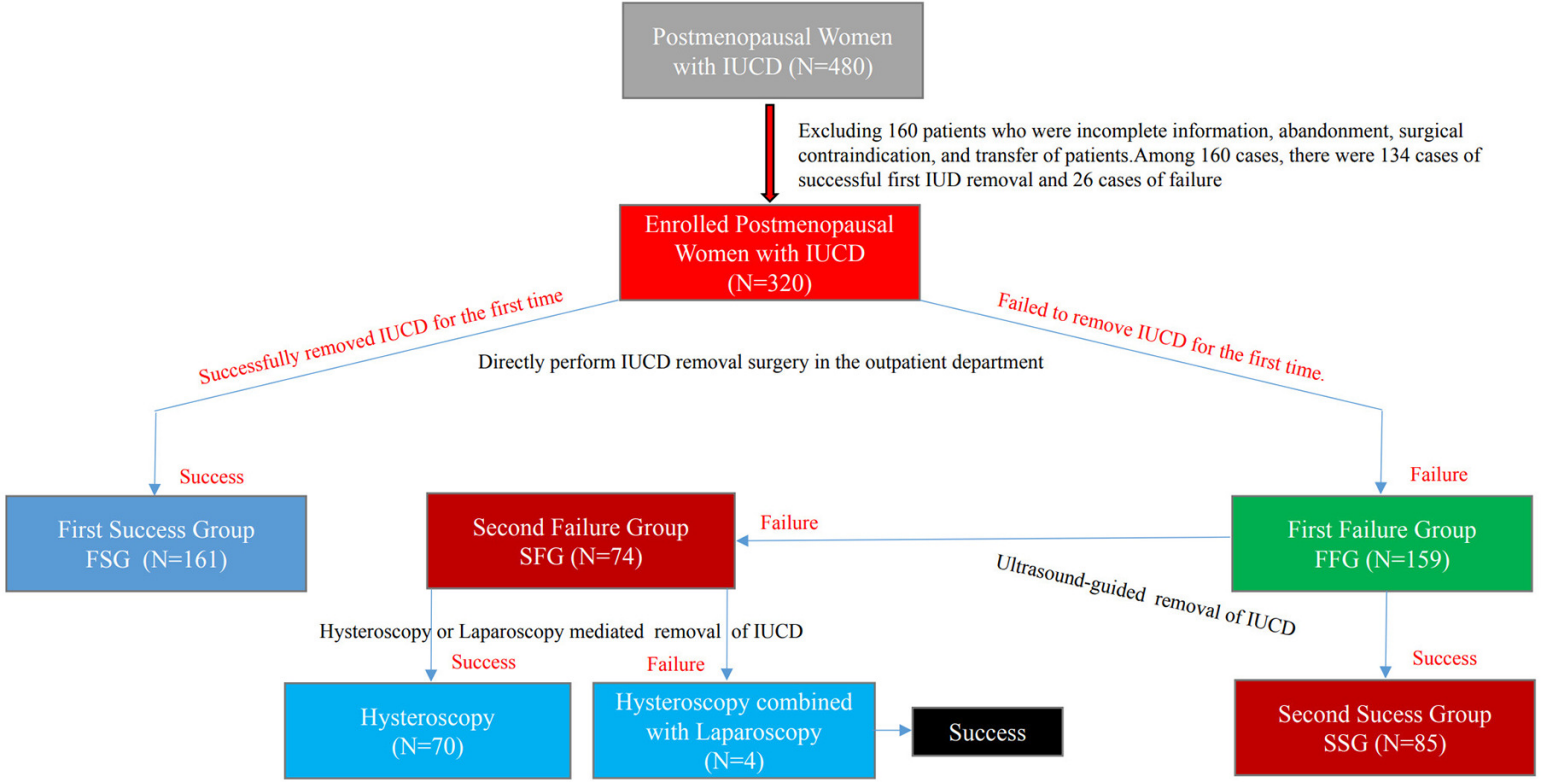
Grouping and handling flow chart of enrolled patients.

Of the 480 patients, 160 were excluded because of incomplete information, surgery abandonment, surgical contraindication, or transfer midway through treatment. In total, 320 patients were enrolled in this study. Based on the results of, and strategy for, removal of IUCD, the 320 patients were grouped either into the First Success Group (FSG, *N* = 161) or the First Failure Group (FFG, *N* = 159). The 159 in the First Failure Group were then further classified into either the Second Success Group (SSG, *N* = 85) or the Second Failure Group (SFG, *N* = 74). Of the 74 patients in the SFG, 70 achieved success under hysteroscopy, while four achieved success after combined laparoscopy. The specific disposal process of selected objects is shown in Figure 1.

### 3.2 The baseline characteristic of the 320 enrolled postmenopausal women

**Figure 2:**
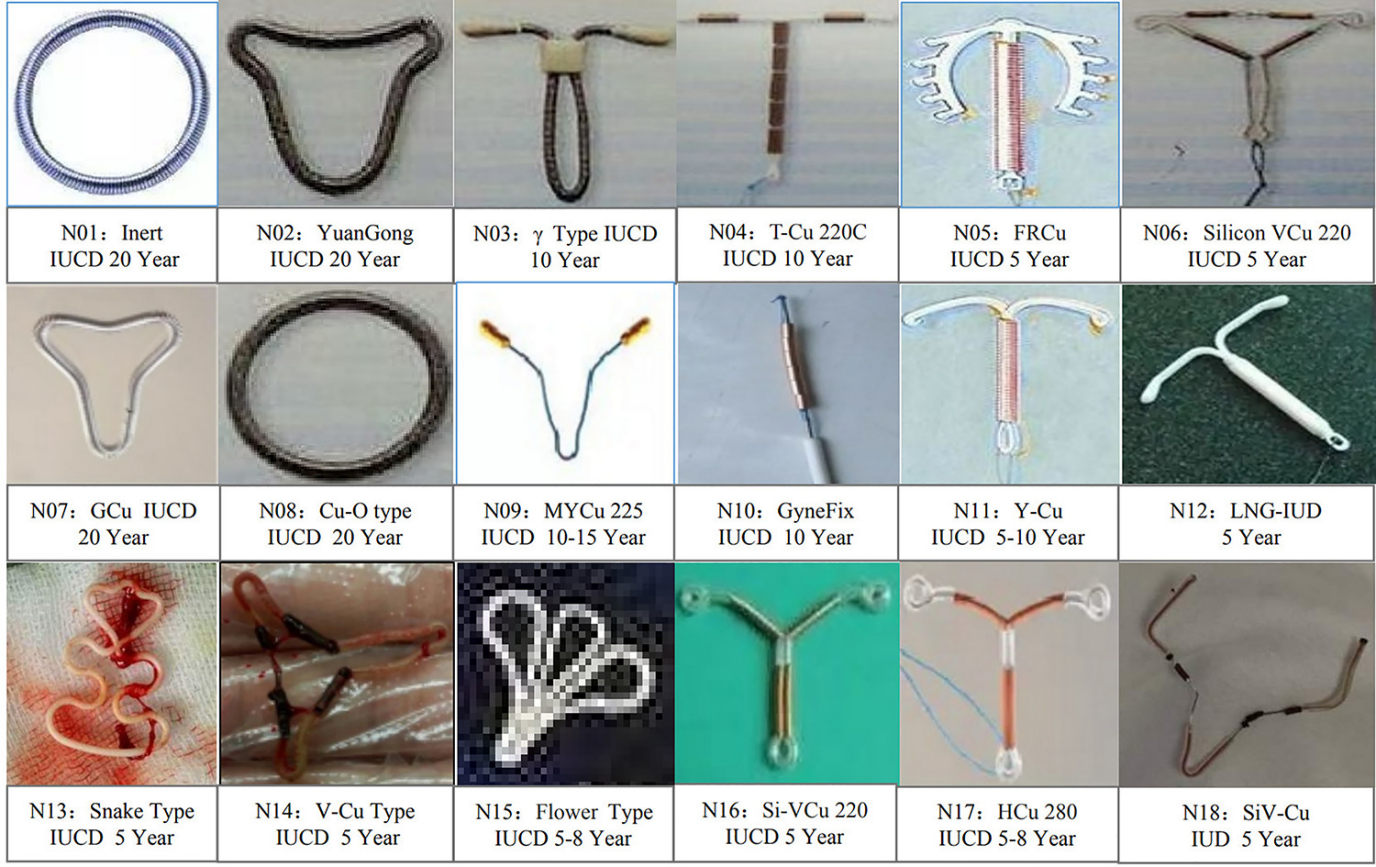
Introduction to the types and service life of IUCD involved in this study.

The data in Table 1 show that the failure rate of a first removal of an IUCD was as high as 49.69% (159/320). It also shows that 160 patients were excluded due to incomplete data (134 first successful cases and 26 first failed cases). In fact, the overall failure rate of a first removal of an IUCD among the 480 selected patients was 38.53% (185/480). By using different statistical methods, it was found that, except for BMI, parity, metal condition of IUCD, abortion time, and surgical qualification, all other variables, including age, duration of menopause, IUCD duration, uterine surgery history, education level, insertion time of IUCD, the condition of the tail silk string of the IUCD, IUCD shape, IUCD position, preoperative IUCD type of patient, gross uterine size, uterine position, cervical size, and cervical canal external opening, showed significant statistical significances between the two groups, with *P*-values less than 0.05.The above results are shown in Table 1. In addition, among the commonly used IUCDs, metal-containing IUCDs account for a higher proportion (93.13%), including from N01 to N12, and the first four types, from N01 to N04, are the most common (shown in Figure 2).

**Table 1:** The baseline characteristics of the enrolled 320 postmenopausal women.

Variables	First success group (FSG) (*N* = 161)	First failure group (FFG) (*N* = 159)	*P*-value
Age (year)	54.66 ± 3.04	58.23 ± 6.00	0.000^c^
BMI (kg/m^2^)	22.56 (20.01–28.09)	23.03 (19.20–26.50)	0.151^b^
Duration of menopause (year)	5 (1–12)	8 (1–30)	
≦5	101	35	0.000^b^
5–10	51	61	
≧10	9	63	
Abortion time (number)	3 (1–4)	3 (1–5)	0.000^d^
Parity (number)	2 (1–2)	2 (1–3)	0.789^d^
IUCD retention time (year)	18 (5–32)	21 (8–35)	
≦5	38	7	0.000^b^
5–10	93	64	
≧10	30	88	
Uterine surgery history (number)			
No surgery history	125 (77.64%)	96 (60.38%)	0.000^a^
Surgery history	38 (23.60%)	61 (38.36%)	
Myomectomy/Cesarean section	18 (11.18%)	14 (8.81%)	
Cervical conization/LEEP	20 (12.42%)	47 (29.56%)	
Education level (number)			
Illiterate/Primary/Junior/High	121 (75.16%)	141 (88.68%)	0.000^a^
University	40 (24.84%)	18 (11.32%)	
Insertion time of IUCD			
Convention	98 (60.87%)	48 (30.19%)	0.000^a^
After induced abortion	58 (36.02%)	95 (59.75%)	
After cesarean section	5 (3.11%)	16 (10.06%)	
Preoperative IUCD type of patients			
Known	88 (54.66%)	109 (68.55%)	0.014^a^
Unknown	73 (45.34%)	50 (31.45%)	
String condition of IUCD			
Yes	35 (21.74%)	18 (11.32%)	0.006^a^
No	126 (78.26%)	141 (88.68%)	
Metal condition of IUCD			
Yes	148 (91.93%)	150 (94.34%)	0.410^a^
No	13 (8.07%)	9 (5.66%)	
IUCD shape by imaging examination			
Ring + V-type	96 (59.63%)	66 (41.51%)	0.000^a^
T + γ + Y-type	49 (30.43%)	23 (14.47%)	
Unknown/Other type	16 (9.94%)	70 (44.03%)	
IUCD position			
Normal	139 (86.34%)	65 (64.72%)	0.000^a^
Embedded	22 (13.66%)	72 (45.28%)	
Gross uterine size			
Normal sized uterus	90 (55.90%)	39 (24.53%)	0.000^a^
Moderate atrophic uterus	50 (31.06%)	27 (16.98%)	
Severe atrophic uterus	2 1 (12.96%)	93 (58.49%)	
Uterine position			
Anterior/Horizontal/Posterior	111 (68.94%)	67 (42.14%)	0.000^a^
Anterior/Posterior flexion	50 (31.06%)	65 (40.88%)	
Unknown	0 (0.00%)	27 (16.98%)	
Cervical size			
Normal size/Mild	91 (56.52%)	39 (24.53%)	0.000^a^
Moderate cervical atrophy	47 (29.19%)	25 (15.72%)	
Severe cervical atrophy	23 (14.29%)	95 (59.75%)	
Cervical canal external opening			
Clearly visible	121 (75.16%)	49 (30.82%)	0.000^a^
Needle-shaped visible	40 (24.84%)	104 (65.41%)	
Suspicious visible	0 (0.00%)	6 (3.77%)	
Surgical qualification			
Attending physician	70 (43.48%)	89 (55.97%)	0.063^a^
Associate chief physician	14 (8.70%)	12 (7.55%)	
Chief physician	77 (47.83%)	58 (36.48%)	

Nonnormally distributed data are presented as the median (M) and interquartile range (Q), the mean ± standard deviation is used for continuous quantitative data. The rate and frequency of counting data are presented as N%. Convention: IUCD should be placed 3–5 days after clean menstruation (10). String condition of IUCD: With or without tail wire when removing IUCD. Surgical qualification: The physicians involved in this study are all gynecologists with different professional titles.

^a^ P: Pearson Chi-square test.

^b^ P: Mann–Whitney *U*-test.

^c^ P: Independent-Sample *T*-test.

^d^ P: Kolmogorov–Smirnov Test.

### 3.3 Spearman correlation analysis between risk factors that pass the initial screening

Spearman correlation analysis showed that age is positively correlated with both the length of menopause (*r* = 0.821, *P* = 0.000) and the duration of IUCD (*r* = 0.516, *P* = 0.000). There was a significant interaction factor between only the age and the length of menopause, which is expected, as older postmenopausal women naturally have a longer menopausal period. This correlation may reduce the accuracy of the Logistic Regression analysis. However, as there was no additive interaction between age and menopausal duration or IUCD retention time, age does not appear to be the primary factor affecting the success or failure of IUCD removal (11, 12). In addition, there was no significant correlation between the other selected variables, as all absolute values of *r* were less than 0.5. There was only a relatively weak correlation between the preoperative patient's awareness of the type of IUCD and their educational level (*r* = 0.363, *P* = 0.000).

### 3.4 Multivariable logistic regression analysis of risk parameters related to the first removal of an IUCD in postmenopausal women in outpatient clinics

After gradually stratifying the risk variables and conducting multivariable Logistic Regression Analysis, it was found that 13 risk variables were identified, and among them, eight risk factors were used in this study based on statistical significance (all *P*-value <0.1). These were duration of menopause (DM), IUCD retention time (IUCD-RT), uterine surgery history (USH), preoperative IUCD type (P-IUCD-T), the IUCD shape confirmed by ultrasound (IUCD-S), IUCD position (IUCD-P), gross uterine size (GUS), and uterine position (UP). The above results are shown in Table 2.

**Table 2:** Multi-variable logistic regression analysis of risk parameters related to the removal of IUCDs in postmenopausal women for the first time in outpatient clinics.

Parameter	B	SE	Wald/χ^2^	Exp(B)/OR	*P*-value
DM	0.765	0.267	8.225	2.149	0.004
IUCD-RT	0.641	0.312	4.223	1.899	0.040
IUCD-IM	0.352	0.314	1.254	1.422	0.263
USH	0.894	0.243	13.545	2.445	0.000
EL	0.346	0.542	0.408	1.414	0.523
P-IUCD-T	0.701	0.419	2.799	2.016	0.094
TSC	0.372	0.423	0.771	1.450	0.380
IUCD-S	0.761	0.240	8.885	2.046	0.003
IUCD-P	0.931	0.475	3.847	2.536	0.050
GUS	0.856	0.426	4.040	2.353	0.044
UP	0.821	0.274	8.990	2.272	0.003
GCS	0.274	0.407	0.452	1.315	0.501
CCEO	0.632	0.437	2.087	1.881	0.149
Constant	−5.442	0.756	51.469	0.004	0.000

SE, standard error; DM, duration of menopause; IUCD-RT, IUCD retention time; IUCD IM, IUCD insertion time; USH, uterine surgery history; EL, education level; P-IUCD-T, preoperative IUCD type; TSC, tail string condition; IUCD-S, IUCD shape by ultrasound; IUCD-P, IUCD position; GUZ, Gross uterine size; UP, uterine position; GCS, gross cervical size; CCEO, cervical canal external opening.

### 3.5 The results of multivariable binary logistic stepwise regression analysis of risk parameters related to the removal of IUCDs and modeling a scoring system based on a different regression coefficient

After multivariate binary logistic stepwise regression analysis, it was found that 7 high-risk variables were identified for the first failure to remove an IUCD, namely duration of menopause, IUCD retention time, uterine surgery history, the shape of IUCD as confirmed by ultrasound, IUCD position, gross uterine size, and uterine position. The Homer–Lemeshow goodness-of-fit test of the model showed great significance (χ^2^ = 236.558, *P* = 0.000). In addition, through the RSSR-IUCDs, the specific scoring values for each patient can be calculated and obtained. The actual score value was rounded to the nearest whole number, based on the principle of minimum regression coefficients and rounding to set the minimum regression coefficient as the base in this study.. Taking integer values yields the corresponding scores for different high-risk factors. The score range of RSSR-IUCDs (Minimum–Maximum) is 0–40. The above results are shown in Table 3.

**Table 3:** The results of multi-variable binary logistic stepwise regression analysis and scoring system based on a different regression coefficient (step 7, total score = 40 points).

Parameter	RC	SE	Wald/χ^2^	OR	95% CI	*P*-value	Scoring
Duration of menopause (year)							
≦5	–	–	–	1	Reference		
5–10	0.841	0.393	4.587	2.318	1.074–5.004	0.032	**3**
≧10	1.685	0.583	8.353	5.394	1.720–16.916	0.004	**6**
IUCD retention time (year)							
≦5	–	–	–	1	Reference		
5–10	0.607	0.598	1.031	1.836	0.568–5.930	0.310	**3**
≧10	1.453	0.651	4.976	4.275	1.193–15.322	0.026	**5**
Uterine surgery history							
No surgery	–	–	–	1	Reference		
CS/Myomectomy	0.941	0.410	5.270	2.563	1.147–5.732	0.022	**3**
CC/LEEP	1.928	0.503	14.721	6.877	2.568–18.413	0.000	**6**
The shape of IUCD by ultrasound							
Ring and V	–	–	–	1	Reference		
T, γ, and Y	0.309	0.441	0.490	1.951	0.573–3.236	0.484	**1**
Unknown/other	1.276	0.454	7.881	4.136	1.470–8.726	0.005	**4**
IUCD position							
Normal	–	–	–	1	Reference		
Embedded	1.195	0.447	7.139	3.305	1.375–7.943	0.008	**4**
Gross uterine size							
Normal/Mild-AU	–	–	–	1	Reference		
Moderate-AU	1.083	0.409	7.031	2.954	1.327–6.580	0.008	**4**
Severe-AU	2.505	0.501	24.963	12.243	4.583–32.707	0.000	**8**
Uterine position							
Normal	–	–	–	1	Reference		
Flexion	0.418	0.452	0.856	1.520	0.626–3.687	0.355	**2**
Unknown	2.215	0.689	10.332	9.158	2.372–35.342	0.001	**7**
Constant	**−4** **.** **192**	**0** **.** **710**	**34** **.** **851**	**0** **.** **015**		**0** **.** **000**	

IUCD, intrauterine contraceptive device; RC, regression coefficient; SE, standard error; OR, odds ratio; CI, confidence interval; CC, cervical conization; LEEP, loop electrosurgical excision procedure. Flexion: The state of extreme anterior or posterior curvature of the uterus. Scoring: Set the minimum regression coefficient as the base, and the ratio of each regression coefficient to it, rounded to the nearest whole number. The bold values in the table are the final scores after rounding. The gross uterine size obtained through gynecological palpation includes normal, mild atrophy, moderate atrophy, and severe atrophy.

### 3.6 The clinical value of RSSR-IUCDs in predicting the first failure to remove an IUCD in the FFG

**Figure 3:**
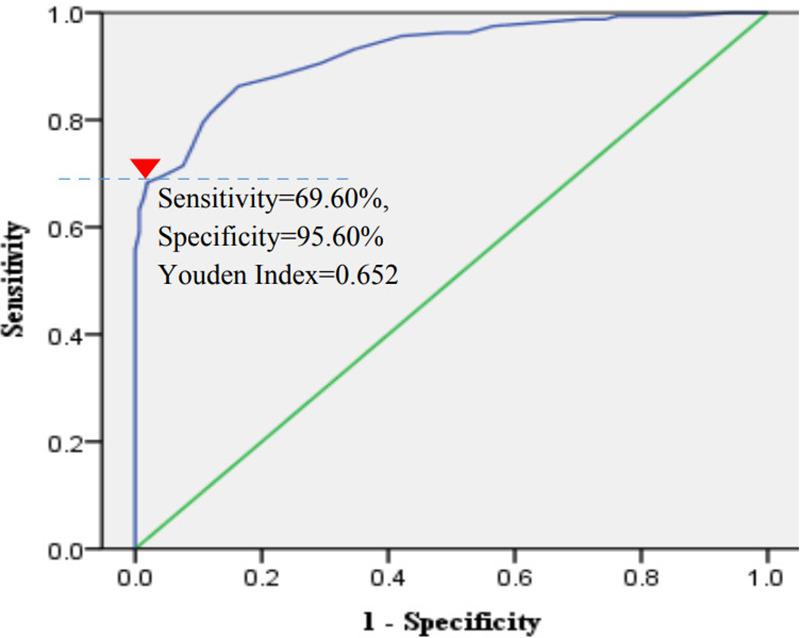
The ROC curve of RSSR-IUCDs.

The ROC curve shows that the AUC of ROC is 0.926, SE is 0.014, and the 95% confidence interval is 0.898–0.953 (*P* = 0.000). When the ideal cut-off value determined by analysis of the ROC curve of RSSR-IUCDs in predicting the first failure to remove IUCD is 20 points, the sensitivity, specificity, and Youden index are 69.60%, 95.60%, and 0.652 respectively. The definition of the ideal cutoff value is determined based on the difference between the horizontal and vertical coordinates being equal to the maximum diagnostic accuracy, which is the maximum Youden index (displayed at the red inverted triangle mark in Figure 3).

### 3.7 Comparison and analysis of the distribution of reasons for the failure to remove an IUCD between the FFG and SFG

Among the 159 patients who underwent a second attempt at IUCD removal under ultrasound guidance, the failure rate was 46.54% (74/159). There was no significant difference compared to the first attempt failure rate of 49.69% (159/320) without ultrasound guidance (χ² = 0.013, *P* = 0.911), as determined by the Chi-square test. However, the cumulative failure rates were, FFG 49.69% (159/320) and SFG 23.13% (74/320), with significant differences. By further analyzing the distribution of reasons for the failure to remove IUCDs, it was found that “embedding” related to the IUCD and “ probe unable to enter uterine cavity” related to the uterus were relatively more common in the failure to remove IUCDs for the first time, accounting for 40.88% (65/159) and 22.01% (35/159), respectively. Less common reasons included “residue” and “uterine perforation”, which accounted for 5.66% (9/159) and 2.52% (4/159). Among the reasons for the second failure to remove IUCD, “embedding” and “rupture” related to IUCD accounted for 48.65% (36/74) and 24.32% (18/74), respectively. In addition, the first failure rates of “uterine perforation” and “cervical adhesion” among uterine-related reasons were 2.52% (4/159) and 7.55% (12/159), respectively, and the second failure rates were both 5.41% (4/74). The above results are shown in Table 4.

**Table 4:** Analysis of the distribution of reasons for the failure to remove IUCD.

Reason	IUCD-related reasons				Uterine-related reasons		
	Embedding	Fracture	Probe unable to detect	Residue	Uterine perforation	Cervical adhesions	Probe unable to enter uterine cavity
The first failure to remove IUCD without ultrasound mediation (*n* = 159)							
Case number	65	22	12	9	4	12	35
Proportion (%)	40.88%	13.83%	7.55%	5.66%	2.52%	7.55%	22.01%
The second failure to remove IUCD with ultrasound mediation (*n* = 74)							
Case number	36	18	2	6	4	4	4
Proportion (%)	48.65%	24.32%	2.70%	8.11%	5.41%	5.41%	5.41%

### 3.8 Analysis of the distribution of different reasons for the second failure to remove an IUCD

An in-depth statistical analysis of the reasons for failure in the 74 patients who were unsuccessful in the second IUCD removal attempt—particularly in relation to different IUCD types—revealed that the main cause was IUCD embedding, accounting for the highest failure rate at 48.64%. This was followed by IUCD rupture at 25.68% (19/74) and IUCD residue at 17.57% (13/74).The type distribution of IUCDs are mainly reflected in N04 (TCu-220C), N03 (γ-type), N05 (FRCu), and N02 (YuanGong), with 13.21%, 5.03%, 5.03%, and 3.77%, respectively. In addition, the types of IUCD with the highest incidence of implantation, rupture, and residue are all N04 (TCu-220C). The proportion of service life of exceeding IUCDs is as high as 83.78% (62/74). The above results are shown in Table 5 and Figure 2.

**Table 5:** Further analysis of the distribution of different reasons related to different types of IUCD and uterine perforation for the second failure to remove IUCD.

IUCD type	Ring + V-type								T + γ + Y-type							Unknown/Other type				
	N01	N02	N07	N08	N06	N09	N14	N18	N03	N04	N05	N11	N12	N16	N17	N10	N13	N15	UK	Total
Embedding	2	5	3	2	1	5	2	0	3	7	3	1	0	0	0	0	2	0	0	36
Fracture	1	1	1	1	1	0	1	0	3	4	3	1	0	0	0	0	1	0	1	19
PUD	0	0	0	0	0	0	0	1	0	0	0	0	0	0	0	0	0	0	1	2
Residue	0	0	0	0	0	0	0	0	2	6	2	0	0	0	0	2	1	0	0	13
UP	0	0	0	0	0	0	0	0	0	4	0	0	0	0	0	0	0	0	0	4
Total^a^	3	6	4	3	2	5	3	1	8	21	8	2	0	0	0	2	4	0	2	74
SFP (%)	4.05	8.12	5.41	4.05	2.70	6.76	4.05	1.35	10.81	28.38	10.81	2.70	0.00	0.00	0.00	2.70	5.41	0.00	2.70	100.00
Total^b^	9	11	10	12	12	6	4	2	23	20	5	4	10	2	4	12	6	2	5	159
FFP (%)	5.66	6.92	6.28	7.55	7.55	3.77	2.52	1.26	14.47	12.58	3.14	2.52	6.28	1.26	2.52	7.55	3.77	1.26	3.14	100.00

PUD, probe unable to detect; UP, uterine perforation; SFP, second failure proportion of different type IUCD; FSP, first failure proportion of different type IUCD; UK, unknown.

^a^ Total, the total number of different IUCD types in second failure to remove IUCD.

^b^ Total, the total number of different IUCD types in first failure to remove IUCD.

## 4 Discussion

The IUCD, as a long-active reversible contraceptive, is continually being used as an effective device for preventing pregnancy, especially in economically underdeveloped and rural areas in China (2). According to literature reports, from 1982 to 1990 alone, nearly 10 million women in China were equipped with an IUCD (3). The complications caused by the removal of an IUCD is one of the major clinical practical issues currently faced by Chinese gynecologists. Complications from removal of IUCDs may include, but are not limited to, embedment or fragmentation (6), residue (6, 13, 14), and uterine perforation (9, 15). Especially for postmenopausal women in rural hospitals in China, the complications caused by the removal of IUCDs are becoming increasingly prominent. In this study, we found that the failure rate of first IUCD removal was as high as 49.69% (159/320), which is inconsistent with the reported 36.38% in the literature (4). Through analysis of the reasons for higher failure rates of first IUCD removal, we revealed that it was related to the inclusion of the study population. Considering that 134 cases patients with a successful first IUCD removal were excluded from 480 cases due to incomplete medical history, incomplete surgical records, and referrals (showed in Figure 1), the actual failure rate of first IUCD removal should be 38.54% (185/480); this was roughly consistent with literature reports (4).

Univariate analysis identified 15 risk factors associated with the first attempt at IUCD removal. Among these, age was significantly correlated with both the length of menopause (*r* = 0.821, *P* = 0.000) and IUCD retention time (*r* = 0.516, *P* = 0.000), as determined by Spearman correlation analysis. Although results seemed to confirm the risk of removing IUCD increased with age in clinical practice (3, 4), it is suggested in fact that age as a risk factor for initial screening plays a role by influencing menopause length and IUCD retention time (4). In addition, there is no obvious correlation between menopause length and IUCD retention time (absolute value of all *r*-value <0.25). Therefore, age was excluded in this study because of the lack of an additive interaction between age and menopausal duration or IUCD retention time, and lack of an additive interaction has an impact on the reliability and statistical valence of multi-variable Logistic Regression Analysis results according to principles of statistical treatment (11, 12). Finally, Multivariate Binary Logistic Stepwise Regression Analysis displayed that seven high-risk factors were included in the construction of the research model named RSSR-IUCDs. Among them, menopause length, IUCD retention time, uterine surgery history, uterine position, and gross uterine size were key risk factors for failure to remove IUCDs for the first time and the highest proportion of scores was 30% (12/40). Numerous clinical practices (3, 4) have confirmed that menopause length and IUCD retention time are the main risk factors for IUCD removal failure. However, as of now, there have been few relevant literature reports on uterine surgery history and uterine position (16). We speculate that a history of uterine surgery may increase the chances of IUCD implantation (17) while also increasing the risk of adhesions in the uterine cavity and/or cervical canal. Adhesions in the uterine cavity and cervical canal, as well as the abnormal position of the uterus, may increase the difficulty for probes to enter the uterine cavity smoothly. According to literature reports, the size of the uterine cavity is one of the high-risk factors for IUCD implantation (18, 19), which also directly confirms the results of this study, although it was difficult to estimate the gross uterine size through gynecological palpation in this study. IUCD embedment also proved to be a high-risk factor for failure to remove an IUCD in this study. Although ultrasound evaluation of IUCD implantation has good clinical value, its limitations are also obvious (5, 18, 19). In this study, we found that preoperative ultrasound evaluation considered 65 patients with IUCD implantation, but after another intraoperative ultrasound evaluation, only 36 patients had their implantation confirmed; this indicated that the evaluation of IUCD implantation by ultrasound is also influenced by other factors, such as the experience and technical level of ultrasound physicians, resolution of ultrasound equipment of different hospitals, and the degree of abdominal wall hypertrophy of the examinee. However, three-dimensional ultrasounds may offer higher accuracy and reliability in evaluating uterine cavity size and IUCD position in postmenopausal women (19, 20), although this is also limited by the economic and equipment conditions of primary hospitals. Some studies have also confirmed that CT detection may be the most reliable diagnostic method for evaluating the location of IUCDs (21), but its cost is a barrier in rural hospitals. Based on the above analysis and literature reports, theoretically, constructing RSSR-IUCDs is in line with clinical practice and economic foundations. Our data also show that the total score is 40 points. When the ideal cut-off value was 20 according to the ROC described in Figure 3, the sensitivity, specificity, and Youden index of prediction of the first failure without ultrasound-guided removal of IUCDs were 69.60%, 95.60%, and 0.652, respectively. Its specificity will provide enormous evaluation value for clinical practices. However, in this study, we also found that there was no statistically significant difference in the failure rate of 159 cases of second IUCD removal under ultrasound guidance (46.54%) compared to the failure rate of first IUCD removal under non-ultrasound guidance (49.69%). This seems to be inconsistent with the literature reporting that ultrasound mediation can reduce the risk of failure in removing IUCDs (22). However, overall, there is a significant difference in the cumulative failure rate of IUCD removal under ultrasound mediation compared to the failure rate of IUCD removal under non-ultrasound mediation.

Further analysis of the reasons for failure during the second IUCD removal revealed that IUCD embedding, rupture, and residue accounted for a relatively large proportion of cases: 48.64% (36/74) for embedding, 25.68% (19/74) for rupture, and 17.57% (13/74) for residue. The second IUCD removal under ultrasound guidance in clinical practice cannot completely resolve certain reasons caused by the first failure, such as IUCD rupture, embedding, residue, and uterine perforation. It is not difficult to notice that ultrasound mediation can only solve certain causes of FFG, such as “cervical adhesion” (7.55%) and “probe failure to enter the uterine cavity” (22.01%). The reason for this may be closely related to exceeding the service life of IUCDs (83.78%) and blindly failing to remove the ring for the first time without adequate preoperative risk assessment.

Undoubtedly, the shape of an IUCD is also an important risk factor (17), although this study found that its impact seems to be minimal (0–4 points). This may be related to the large proportion of IUCDs from N01 to N03, which is also in line with China's national conditions. In addition, by further analysis of the distribution of different reasons related to IUCD and uterine perforation for the second failure to remove an IUCD (showed in Tables 4, 5), we found that N04 (TCu-220C) is the primary cause of implantation, rupture, residue, and uterine perforation, which is consistent with literature reports (23, 24). However, some studies have also found that “V”-shaped IUCDs are the main type to cause perforation and implantation (17). The reason for this may be closely related to exceeding the service life of IUCDs and failing to remove them the first time. This once again confirmed the clinical predictive value of RSSR-IUCDs. The construction of the RSSR-IUCDs system reduced the risk of failure and unnecessary complications of blind removal of IUCD to some extent in postmenopausal women. The RSSR-IUCDs also provide a reliable basis for the management strategy of removing IUCDs in postmenopausal women. In addition, the selective implementation of ultrasound-guided removal of IUCDs through RSSR-IUCDs may reduce the costs of implementing the national family planning policy, especially by reducing the cost of removing IUCDs for postmenopausal women in rural hospitals in China. However, this requires further multi-center, large-scale prospective studies and related economics research.

However, the limitations of this study are also evident; for example, some risk factors included subjective evaluation indices, such as the grading of uterine and cervical atrophy. Currently, there is no objective basis standard, and it is only limited to clinical practice evaluation. This may raise some doubts about the reliability of the results of this study. If ultrasound is introduced to quantitatively evaluate the size and position of the uterus and cervix, it will greatly improve the reliability and objectivity of experimental data to a certain extent. However, in rural hospitals there is still a need to increase standardized training on ultrasound evaluation of IUCDs.

## 5 Conclusions

The scoring system RSSR-IUCDs is a scientific, reasonable, and clinically feasible evaluation system and management strategy for removing IUCDs in postmenopausal women, which is expected to become a guiding scoring system in accordance with clinical practice for postmenopausal women before the removal of IUCDs in China.

## Data Availability

The original contributions presented in the study are included in the article/Supplementary Material; further inquiries can be directed to the corresponding author.
